# Racial Disparities in Cognitive Function: The Roles of Cumulative Stress Exposures Across the Life Course

**DOI:** 10.1093/geroni/igaa057.1627

**Published:** 2020-12-16

**Authors:** Ruijia Chen, Jennifer Weuve, Laura Kubzansky, David Williams

**Affiliations:** 1 Harvard University, Cambridge, Massachusetts, United States; 2 Boston University, Boston, Massachusetts, United States; 3 Harvard T.H. Chan School of Public Health, Boston, Massachusetts, United States

## Abstract

Introduction: Racial disparities in cognitive function have been well-documented in the literature, but factors driving the disparities remain under explored. This study aims to quantify the extent to which cumulative stress exposures across the life course explain Black–White disparities in executive function and episodic memory. Method: Data were drawn from the 2004–2006 wave of the Midlife Development in the United States (MIDUS) and the MIDUS refresher study (N=5,967, 5,277 White, 690 Black). Cumulative stress exposures were assessed by using 10 domains of stressors (e.g., financial stress, childhood adversity). Cognitive function was assessed using the Brief Test of Adult Cognition by Telephone. Marginal structural models were conducted to quantify the proportion of the effect of race/ethnicity status on cognitive function that can be explained by cumulative stress exposures. Result: Blacks reported higher levels of cumulative stress exposures and lower average levels of executive function and episodic memory than Whites. Cumulative stress exposures explained 8.43% of the disparities in executive function and 13.21 % of the disparities in episodic memory. Cumulative stress exposures had stronger effects on racial disparities in cognitive function in the older age group (age≥ 55 years old) than in the younger age group (age < 55 years old). Conclusion: Cumulative stress exposures explain modest proportions of racial disparities in levels of cognitive function. Interventions that focus on reducing stress exposures or improving coping resources among Blacks may help lessen racial disparities in cognitive function at the population level.

